# Calendar

**DOI:** 10.1155/S146392467900050X

**Published:** 1979


Additional features include, computer-assisted method calibration, automatic instrument standardization, instrument diagnostic programs, expanded report format, and alphanumeric keyboard and display.

Operator efficiency is greatly increased. The 'aca' III has the capacity to allow new methods to be added as they are developed by Du Pont. Vital parameters are displayed in a selected language to aid instrument operation. The instrument is compatible with any on-line laboratory information system.
*Du Pont de Nemours SA, rue de la Fusee 100, Mercure Centre, 1130 Brussels, Belgium.*

### New methodologies

A number of new diagnostic methods and program cards are now available on the Vitatron PA800 programmable analyser. Software has now been written to enable the user to assay immunoglobulins by the Boehringer Tinaquant methods, coagulation factors, as produced by Kabi and Boehringer and an extended number of EMIT procedures, as produced by Syva. A water filled reagent dispensing system, which requires no priming, ensures minimal expenditure on diagnostics and the programmable Canon calculator process data and produces hard copy data in real concentration terms, from typically non-linear calibration curves.
*MSE Scientific Instruments, Manor Royal, Crawley, West Sussex, RH10 2QQ, UK.*

*Editor's Note:-*
*The address of the manufacturer appears in italic at the end of each item. In some cases this address will be that of a subsidiary to the manufacturing company as the address given is that from which the information has been obtained.*

### Calendar

**Editor's Note:**
*Organisers of conferences, seminars etc. should send details for inclusion in this calandar as soon as the relevant information is available and not later than three months before the event.*

### 1979

**3rd International Symposium on Capillary Chromatography**
April 30-May 3, Hindelang/Allgau, W. Germany.
*R.E. Kaiser, P.O. Box 1308, D-6702 Bad Durkheim 1, Germany.*

**Ninth Annual Symposium on the Analytical Chemistry of Pollutants**
May 7-9, Jekyll Island, U.S.A.
*Mrs. Elaine McGarity, U.S. Environmental Protection Agency, Environmental Research Laboratory, College Station Road, Athens, Georgia 30605, U.S.A.*

**Current Developments in the Clinical Applications of HPLC, GC and MS**
May 30-June 1, Harrow, U.K.
*Symposium Secretariat, Division of Clinical Chemistry, Clinical Research Centre, Harrow, Middlesex, U.K.*

**3rd European Congress of Clinical Chemistry.**
June 3-8, Brighton, U.K.
*Dr. P.J.N. Howarth, Department of Chemical Pathology, Guy's Hospital, Medical School, London SE1 9RT, U.K.*

**2nd World Chromatography Conference**
June 5-6, Lisbon, Portugal.
*V.M. Bhatnagar, P.O. Box 1779, Cornwall, Ontario K6H 5V7, Canada.*

**10th International Symposium on Chromatography and Electrophoresis**
June 19 - 20, Venice, Italy.
*Dr. Alberto Frigerio, Instituto di Riccerche Farmacologiche "Mario Negri," Via Eritrea 62, 20157 Milan.*

**8th International Conference on Atomic Spectroscopy**
July 1 - 6, Cambridge, U.K.
*Association of British Spectroscopists, P.O. Box 109, Cambridge CB1 2HY, U.K.*

**2nd Canadian World Chromatography Conference**
July 5 - 6, Toronto, Canada.
*V.M. Bhatnagar, P.O. Box 1779, Cornwall, Ontario K6H 5V7, Canada.*

**Summer School on Automatic Methods of Analysis**
July 9 - 13, Swansea, U.K.
*D. Porter, Laboratory of the Government Chemist, Cornwall House, Stamford Street, London SE1 9NQ, U.K.*

**Analysis '79: Automation in Industrial and Clinical Chemistry.**
July 16 - 18, London, U.K.
*Scientific Symposia Ltd., 33-35 Bowling Green Lane, London EC1R 0DA, U.K.*

**Automatic methods in connection with Trace-organic Analysis**
September 4 - 7, Guildford, U.K.
*Dr. E. Reid, Wolfson Bioanalytical Centre, University of Surrey, Guildford GU2 5XH, U.K.*

**Automation and Computerisation in the Medical Laboratory**

September 5 - 7, Stirling University, Scotland.

*G.W. Thomson, Secretary, IMLS Glasgow Branch, 67 Glen Ogilvie, St. Leonards, East Kilbride, Scotland.*

**International Conference on Flow Analysis**
September 11 - 13, Amsterdam, The Netherlands.
*Secretary FA-Amsterdam, Laboratory for Analytical Chemistry, University of Amsterdam, Nieuwe Achtergracht 166, 1018 WV Amsterdam, The Netherlands.*

**Communications in Microprocessor Industrial Instrumentation**
September 12 - 13, London U.K.
*Mrs. P. Keiller, Sira Institute, South Hill, Chislehurst, Kent BR7 5EH, U.K.*

**Ninth International Meeting on Organic Geochemistry**
September 17 - 20, Newcastle-upon-Tyne, U.K.
*Dr. A.G. Douglas, Organic Geochemistry Unit, Geology Department, Drummond Building, The University, Newcastle-upon-Tyne, NE1 7RU, U.K.*

**Le Chromatographe Automatique Industriel en Lague: Analyseur Sophistique ou Capteur Industriel?**
October 3 - 5, Arles, France.
*Institute de Regulation et Automation Guy Berthier, Chemin des Moines, 13644, Arles, France.*

**Real-time Datahandling and Process Control**
October 23 - 25, Berlin, West Germany
*Congress Organisation Company, John Foster Dulles Allee 10,D-1000 Berlin,West Germany.*

### 1980

**Automation at SAC 80**
July 20 - 26, Lancaster, U.K.
*The Secretary, Analytical Division, The Chemical Society, Burlington House, London, W1Y 0BN, U.K.*

### 1981

**Euroanalysis IV**

August 23 - 28, Helsinki, Finland.

*Association of Finnish Chemical Societies. Executive Secretary, Pohj, Hesperiankatu 3B10, SF-00260 Helsinki 26, Finland.*

